# Supplementary material for: A Systematic Method for the Identification of Aporphine Alkaloid Constituents in Sabia schumanniana Diels Using UHPLC-Q-Exactive Orbitrap/Mass Spectrometry
Source: Molecules. 2022 Nov 7;27(21):7643. doi: 10.3390/molecules27217643 (PMC9656101; doi:10.3390/molecules27217643)
Supplement: Supplementary file 1 [file molecules-27-07643-s001.zip › molecules-1927194-supplementary.pdf]

**Table S1.** The chromatographic and mass data of detected components from *Sabia schumanniana* Diels though UHPLC-Q-Exactive Orbitrap MS.

| Peak | $t_{R(min)}$ | Theoretical Mass *m/z* | Experimental Mass *m/z* | Error (ppm) | Formula $(M+H)^+$or$(M)^+$ | MS/MS fragment (+) | Identification |
|---|---|---|---|---|---|---|---|
| 1 | 3.32 | 358.1649 | 358.1652 | 0.81 | $[C_{20}H_{24}NO_5]^+$ | $MS^2$[358]: 58.0660(100), 255.0667(1), 227.0703(2), 287.0917(4) | $C_{6a}$-hydroxylation of magnoflorine |
| 2 | 4.02 | 490.2072 | 490.2079 | 1.43 | $[C_{25}H_{31}NO_9+H]^+$ | $MS^2$[490]: 297.1122(100), 237.0914(10), 265.0861(85), 328.1546(36) | 11-glc-norisocorydine isomer |
| 3 | 4.05 | 312.1594 | 312.1600 | 1.86 | $[C_{19}H_{22}NO_3]^+$ | $MS^2$[312]: 58.0660(100), 207.0811(1), 267.1014(3) | $C_2$-O-demethylation of magnoflorine isomer |
| 4 | 4.28 | 358.1649 | 358.1656 | 1.84 | $[C_{20}H_{24}NO_5]^+$ | $MS^2$[358]: 58.0659(100), 253.0863(14), 281.0809(40), 285.0740(4), 313.1071(45) | trilobinine isomer |
| 5 | 5.33 | 278.1175 | 278.1178 | 1.20 | $[C_{18}H_{15}NO_2+H]^+$ | $MS^2$[278]:107.0497(100), 246.0928(6), 262.0858(17) | dehydroroemerine |
| 6 | 5.40 | 328.1543 | 328.1546 | 0.81 | $[C_{19}H_{22}NO_4+H]^+$ | $MS^2$[328]:58.0660(100), 177.0551(2), 222.1118(4), 265.0862(17), 283.0967(34) | boldine isomer |
| 7 | 5.71 | 340.1543 | 340.1552 | 2.66 | $[C_{20}H_{22}NO_4]^+$ | $MS^2$[340]: 263.0705(100), 58.0660(13), 235.0756(29), 295.0965(24) | N-methylbulbocapnine isomer |
| 8 | 5.72 | 358.1649 | 358.1651 | 0.56 | $[C_{20}H_{24}NO_5]^+$ | $MS^2$[358]:58.0660(100), 281.0818(4), 285.0774(3), 295.0966(25), 313.1071(22) | trilobinine isomer |
| 9 | 5.77 | 314.1386 | 314.1389 | 0.75 | $[C_{18}H_{19}NO_4+H]^+$ | $MS^2$[314]: 265.0861(100), 58.0660(77), 165.0913(1), 205.0658(1), 237.0910(31), 297.1124(66) | laurolitsine |
| 10 | 5.81 | 298.1437 | 298.1440 | 0.70 | $[C_{18}H_{19}NO_3+H]^+$ | $MS^2$[278]:192.1022(10 | apoglaziovine |

| Peak | $t_{R(min)}$ | Theoretical Mass *m/z* | Experimental Mass *m/z* | Error (ppm) | Formula $(M+H)^+$ or $(M)^+$ | MS/MS fragment (+) | Identification |
|---|---|---|---|---|---|---|---|
| | | | | | | 0), 58.0659(10), 254.0953(19), 283.1197(3) | |
| 11 | 5.83 | 312.1594 | 312.1595 | 0.51 | $[C_{19}H_{22}NO_3]^+$ | $MS^2$[312]: 205.1100(100), 58.0660(19), 207.0819(4), 267.1017(62) | $C_2$-O-demethylation of magnoflorine isomer |
| 12 | 5.88 | 328.1543 | 328.1545 | 0.35 | $[C_{19}H_{22}NO_4+H]^+$ | $MS^2$[328]: 58.0660(100), 237.0907(1), 283.0966(74), 297.1108(3) | boldine isomer |
| 13 | 5.99 | 490.2072 | 490.2076 | 0.98 | $[C_{25}H_{31}NO_9+H]^+$ | $MS^2$[490]: 265.0861(100), 192.1019(15), 237.0901(2), 297.1122(25), 328.1544(32) | 11-glc-norisocorydine isomer |
| 14 | 6.04 | 340.1543 | 340.1545 | 0.52 | $[C_{20}H_{22}NO_4]^+$ | $MS^2$[340]: 263.0704(100), 58.0659(13), 235.0755(29), 295.0962(27) | |
| 15 | 6.05 | 358.1649 | 358.1651 | 0.48 | $[C_{20}H_{24}NO_5]^+$ | $MS^2$[358]: 58.0660(100), 281.0808(6), 285.0773(3), 295.0966(29), 313.1071(28) | N-methylbulbocapnine isomer |
| 16 | 6.22 | 344.1856 | 344.1857 | 0.10 | $[C_{20}H_{26}NO_4]^+$ | $MS^2$[344]: 58.0659(100), 137.0598(30), 143.0493(13), 175.0754(34), 299.1278(12) | trilobinine isomer |
| 17 | 6.30 | 328.1907 | 328.1908 | 0.15 | $[C_{20}H_{26}NO_3]^+$ | $MS^2$[328]: 58.0659(100), 237.0911(3), 252.1143(5), 268.1098(3), 283.1341(26) | zizyphusine+2H |
| 18 | 6.40 | 328.1543 | 328.1544 | 0.26 | $[C_{19}H_{22}NO_4+H]^+$ | $MS^2$[328]: 283.0965(100), 58.0659(25), 237.0913(5), 265.0858(20), 297.1123(14) | N-ring opening-$C_1$-dehydroxylation of magnoflorine isomer |

| Peak | $t_{R(min)}$ | Theoretical Mass *m/z* | Experimental Mass *m/z* | Error (ppm) | Formula $(M+H)^+$or$(M)^+$ | MS/MS fragment (+) | Identification |
|---|---|---|---|---|---|---|---|
| 19 | 6.70 | 342.1700 | 342.1702 | 0.54 | $[C_{20}H_{24}NO_4]^+$ | $MS^2$[342]: 58.0660(100), 237.0903(11), 265.0853(74), 282.0880(13), 297.1115(69) | boldine isomer |
| 20 | 6.79 | 374.1598 | 374.1596 | 0.47 | $[C_{20}H_{24}NO_6]^+$ | $MS^2$[374]: 58.0659(100), 297.1079(1), 329.1034(1) | magnoflorine isomer |
| 21 | 7.04 | 312.1594 | 312.1597 | 1.09 | $[C_{19}H_{22}NO_3]^+$ | $MS^2$[312]: 267.1017(100), 58.0659(39), 207.0814(2) | Di-hydroxylation of magnoflorine |
| 22 | 7.31 | 358.2013 | 358.2012 | -0.32 | $[C_{21}H_{28}NO_4]^+$ | $MS^2$ [358]: 58.0660(100), 281.0813(7), 313.1446(5) | $C_2$-O-demethylation of magnoflorine isomer |
| 23 | 7.38 | 282.1489 | 282.1490 | 0.58 | $[C_{18}H_{19}NO_2+H]^+$ | $MS^2$[282]: 237.0911(100), 58.0660(90), 191.0855(5), 219.0806(23), 251.1063(1) | pareirarinea isomer |
| 24 | 7.44 | 340.1543 | 340.1546 | 0.78 | $[C_{20}H_{22}NO_4]^+$ | $MS^2$[340]: 263.0703(100), 189.0692(2), 217.0644(8), 235.0754(21), 295.0966(42) | lirinidine isomer |
| 25 | 7.53 | 328.1543 | 328.1545 | 0.35 | $[C_{19}H_{22}NO_4+H]^+$ | $MS^2$[328]: 58.0660(100), 178.0864(81), 265.0860(41), 283.0968(43), 297.1123(15) | N-methylbulbocapnine isomer |
| 26 | 7.58 | 358.2013 | 358.2008 | 1.41 | $[C_{21}H_{28}NO_4]^+$ | $MS^2$ [358]: 58.0660(100), 281.0815(7), 313.1059(8) | boldine isomer |
| 27* | 7.58 | 342.1700 | 342.1703 | 0.89 | $[C_{20}H_{24}NO_4]^+$ | $MS^2$[342]: 265.0852(100), 58.0658(21), 282.0877(4), 297.1123(48), 237.0905(4) | pareirarinea isomer |
| 28 | 7.67 | 294.1488 | 294.1491 | 0.76 | $[C_{19}H_{19}NO_2+H]^+$ | $MS^2$[294]: 217.0650(100), | magnoflorine |

| Peak | $t_{R(min)}$ | Theoretical Mass *m/z* | Experimental Mass *m/z* | Error (ppm) | Formula $(M+H)^+$or$(M)^+$ | MS/MS fragment (+) | Identification |
|---|---|---|---|---|---|---|---|
| | | | | | | 58.0659(2), 279.1269(5), 263.0711(5), 250.0958(2) | |
| 29 | 7.68 | 312.1594 | 312.1597 | 0.90 | $[C_{19}H_{22}NO_3]^+$ | MS$^2$[312]: 217.0649 (100), 58.0659(57), 218.0692(7), 237.0875(15), 252.0784(5), 267.1017(15) | dehydronuciferine isomer |
| 30 | 7.69 | 354.1700 | 354.1704 | 1.12 | $[C_{21}H_{24}NO_4+H]^+$ | MS$^2$[354]: 58.0660(100), 251.1074(11), 309.1119(33) | $C_2$-O-demethylation of magnoflorine isomer |
| 31 | 7.72 | 282.1489 | 282.1491 | 0.80 | $[C_{18}H_{19}NO_2+H]^+$ | MS$^2$[282]: 237.0911(100), 58.0660(95), 191.0862(5), 219.0807(24) | N-methyl nantenine |
| 32 | 7.87 | 312.1594 | 312.1597 | 0.90 | $[C_{19}H_{21}NO_3+H]^+$ | MS$^2$[312]: 267.1016(100), 58.0659(56), 217.0650(45), 280.1064(9), 294.1487(9) | lirinidine isomer |
| 33 | 8.04 | 344.1492 | 344.1495 | 0.85 | $[C_{19}H_{22}NO_5]^+$ | MS$^2$[344]: 58.0660(100), 237.0907(6), 265.0860(29), 283.0926(10) | isothebaine isomer |
| 34 | 8.04 | 374.1598 | 374.1603 | 1.49 | $[C_{20}H_{24}NO_6]^+$ | MS$^2$[374]: 58.0659(100), 297.0756(5), 329.1017(43) | N-$CH_3$ -hydroxylation of $C_2$-O-demethylation of magnoflorine |
| 35 | 8.10 | 358.1649 | 358.1653 | 1.23 | $[C_{20}H_{24}NO_5]^+$ | MS$^2$[358]: 313.1071(28), 58.0660(51), 253.0859(4), 281.0804(13), 285.0804(13) | Di-hydroxylation of magnoflorine |
| 36 | 8.35 | 356.1856 | 356.1858 | 0.52 | $[C_{21}H_{26}NO_4]^+$ | MS$^2$[356]: 58.0660(100), 236.0833(3), 264.0785(10), 251.1066(9), 279.1018(79), 280.1082(30), 296.1038(19), | trilobinine isomer |

| Peak | $t_{R(min)}$ | Theoretical Mass *m/z* | Experimental Mass *m/z* | Error (ppm) | Formula $(M+H)^+$ or $(M)^+$ | MS/MS fragment (+) | Identification |
|---|---|---|---|---|---|---|---|
| | | | | | | 311.1280(21) | |
| 37 | 8.42 | 312.1594 | 312.1594 | 0.19 | $[C_{19}H_{21}NO_3+H]^+$ | $MS^2$[312]: 217.0650(100), 58.0659(67), 280.1064(9), 294.1490(23), 296.1046(8), | menisperine isomer |
| 38 | 8.66 | 340.1543 | 340.1548 | 0.87 | $[C_{20}H_{21}NO_4+H]^+$ | $MS^2$[340]: 58.0660(100), 220.0526(2), 264.0755(7), 309.1354(4) | isothebaine isomer |
| 39 | 8.70 | 340.1543 | 340.1546 | 0.69 | $[C_{20}H_{22}NO_4]^+$ | $MS^2$[340]: 263.0703(100), 58.0659(33), 235.0751(3), 295.0965(22) | crebanine |
| 40 | 8.72 | 328.1907 | 328.1906 | -0.24 | $[C_{21}H_{28}NO_4]^+$ | $MS^2$[328]: 58.0659(100), 251.1067(6), 253.1226(2), 283.1328(19) | N-methylbulbocapnine isomer |
| 41 | 8.83 | 400.1755 | 400.1757 | 0.64 | $[C_{22}H_{26}NO_6]^+$ | $MS^2$[400]: 58.0660(100), 295.0961(2), 323.0918(32), 355.1180(29) | N-ring opening-$C_1$-dehydroxylation of magnoflorine isomer |
| 42 | 8.97 | 342.1700 | 342.1702 | 0.63 | $[C_{20}H_{24}NO_4]^+$ | $MS^2$[342]: 58.0659(100), 237.0912(7), 265.0860(49), 282.0894(7), 297.1122(45) | $C_{10}$-$OCH_3$-hydroxylation and $C_{11}$-O-acetylation of magnoflorine |
| 43 | 9.18 | 356.1856 | 356.1857 | 0.27 | $[C_{21}H_{26}NO_4]^+$ | $MS^2$[356]: 58.0660(100), 251.0709(12), 279.1016(66), 280.1085(21), 296.1035(13), 311.0925(18) | magnoflorine isomer |
| 44 | 9.71 | 296.1645 | 296.1646 | 0.45 | $[C_{19}H_{22}NO_2]^+$ | $MS^2$[296]: 251.1068(100), 58.0660(26), 219.0807(24), 221.0957(1), 236.0826(2) | menisperine isomer |
| 45* | 10.29 | 282.1489 | 282.1495 | 0.43 | $[C_{18}H_{19}NO_2+H]^+$ | $MS^2$[282]: 251.1067(100), | $C_1$-demethoxy -$C_2$-dehydrox of |

| Peak | $t_{R(min)}$ | Theoretical Mass *m*/*z* | Experimental Mass *m*/*z* | Error (ppm) | Formula $(M+H)^+$or$(M)^+$ | MS/MS fragment (+) | Identification |
| --- | --- | --- | --- | --- | --- | --- | --- |
| | | | | | | 58.0660(13), 191.0856(5), 219.0806(39) | magnoflorine isomer |
| 46 | 10.32 | 374.1598 | 374.1599 | 0.34 | $[C_{20}H_{24}NO_6]^+$ | $MS^2$[314]: 329.1022(100), 58.0659(14), 297.0758(1) | lirinidine |
| 47 | 11.27 | 294.1488 | 294.1491 | 1.07 | $[C_{19}H_{19}NO_2+H]^+$ | $MS^2$[294]: 217.0650(100), 58.0658(1), 236.0831(1), 250.0946(3), 263.1286(1), 279.1256(8) | di-hydroxylation of magnoflorine |
| 48 | 11.76 | 384.1805 | 384.1812 | 1.64 | $[C_{22}H_{26}NO_5]^+$ | $MS^2$[384]: 325.1070(100), 58.0659(18), 251.1067(4), 279.1019(10), 292.0738(9), 307.0953(4), 339.1230(4) | dehydronuciferine isomer |
| 49 | 12.82 | 356.1856 | 356.1861 | 1.39 | $[C_{21}H_{26}NO_4]^+$ | $MS^2$[356]: 58.0660(100), 251.1067(12), 279.1018(77), 280.1084(27), 296.1043(21), 311.1273(16) | $C_1$-O-acetylation of magnoflorine |
| 50* | 12.86 | 282.1489 | 282.1493 | 1.54 | $[C_{18}H_{19}NO_2+H]^+$ | $MS^2$[282]: 265.1224(100), 58.0659(8), 234.1041(25), 250.0990(60) | menisperine isomer |
| 51 | 12.94 | 266.1176 | 266.1178 | 1.15 | $[C_{17}H_{15}NO_2+H]^+$ | $MS^2$[266]: 131.0494(100), 191.0855(3), 219.0804(14), 249.0912(76) | N-nornuciferine |
| 52 | 13.07 | 294.1489 | 294.1491 | 0.23 | $[C_{19}H_{20}NO_2]^+$ | $MS^2$[310]: 58.0659(100), 191.0862(1), 219.0805(4), 249.0911(55) | anonaine |
| 53* | 13.10 | 280.1332 | 280.1336 | 1.45 | $[C_{18}H_{18}NO_2+H]^+$ | $MS^2$[280]: 249.0912(100), 58.0659(3), 191.0863(3), | roemrefidine |

| Peak | $t_{R(min)}$ | Theoretical Mass *m/z* | Experimental Mass *m/z* | Error (ppm) | Formula $(M+H)^+$or$(M)^+$ | MS/MS fragment (+) | Identification |
|---|---|---|---|---|---|---|---|
| | | | | | | 219.0805(13) | |
| 54 | 13.34 | 292.0968 | 292.0972 | 1.40 | $[C_{18}H_{13}NO_3+H]^+$ | $MS^2$[292]:277.1099(100), 248.0717(8), 264.0999(5) | roemerine |
| 55 | 13.50 | 324.1230 | 324.1235 | 1.56 | $[C_{19}H_{17}NO_4+H]^+$ | $MS^2$[324]: 58.0659(100), 177.0554(3), 263.0940(12), 293.1054(6) | lysicamine isomers |
| 56 | 13.53 | 356.1492 | 356.1496 | 0.99 | $[C_{20}H_{22}NO_5]^+$ | $MS^2$[356]: 58.0659(100), 251.0703(1), 279.1028(7), 311.0918(22) | neolitsine isomer |
| 57 | 13.83 | 312.1230 | 312.1231 | 0.33 | $[C_{18}H_{17}NO_4+H]^+$ | $MS^2$[294]: 295.1328(100), 58.0659(31), 264.1164(52) , 265.0865(21), 280.1095(90) | $C_5$-methylene to ketone of magnoflorine |
| 58 | 13.94 | 312.1594 | 312.1598 | 1.28 | $[C_{19}H_{21}NO_3+H]^+$ | $MS^2$[312]: 295.1329(100), 58.0659(31), 217.0649(90), 280.1095(90), 296.1357(16) | nandigerine |
| 59 | 14.11 | 310.1438 | 310.1441 | 0.97 | $[C_{19}H_{20}NO_3]^+$ | $MS^2$[310]: 58.0659(100), 177.0555(1), 205.0648(3), 237.0909(13), 265.0859(20) | isothebaine isomer |
| 60 | 14.22 | 310.1437 | 310.1440 | 0.87 | $[C_{19}H_{19}NO_3+H]^+$ | $MS^2$[310]: 58.0660(100), 279.1008(1), 264.0792(1), | $C_1$-demethoxy -$C_2$-dehydrox-$C_{10}$,$C_{11}$-Ethyl epoxide of magnoflorine isomer |
| 61 | 14.26 | 296.1645 | 296.1649 | 1.20 | $[C_{19}H_{22}NO_2]^+$ | $MS^2$[296]: 251.1068(100), 58.0660(15), 219.0805(31), 220.0842(3), 221.0957(1), 236.0834(3) | stephanine |
| 62 | 14.61 | 294.1488 | 294.1493 | 1.38 | $[C_{19}H_{19}NO_2+H]^+$ | $MS^2$[294]: 217.0650(100), 58.0660(8), 279.1257(9), 263.1296(1),250.0945( | $C_1$-demethoxy -$C_2$-dehydrox of magnoflorine isomer |

| Peak | $t_{R(min)}$ | Theoretical Mass *m/z* | Experimental Mass *m/z* | Error (ppm) | Formula $(M+H)^+$or$(M)^+$ | MS/MS fragment (+) | Identification |
|---|---|---|---|---|---|---|---|
| | | | | | | 1) | |
| 63 | 15.99 | 294.1124 | 294.1125 | 0.07 | $[C_{18}H_{15}NO_3+H]^+$ | $MS^2$[294]: 262.0863(100), 58.0658(1), 239.0951(1), 257.1901(1) | dehydronuciferine isomer |
| 64 | 17.08 | 310.1438 | 310.1441 | 0.97 | $[C_{19}H_{20}NO_3]^+$ | $MS^2$[310]: 233.0961(100), 58.0661(12), 177.1288(1), 265.1220(23 | N-formyl-annonain |
| 65 | 17.39 | 324.1230 | 324.1231 | 0.33 | $[C_{19}H_{17}NO_4+H]^+$ | $MS^2$[324]: 264.1019(100), 233.0831(22), 263.0940(31), 265.1077(22) | $C_1$-demethoxy -$C_2$-dehydrox-$C_{10}$,$C_{11}$-Ethyl epoxide of magnoflorine isomer |
| 66 | 18.00 | 308.1281 | 308.1283 | 0.49 | $[C_{19}H_{17}NO_3+H]^+$ | $MS^2$[308]: 249.0914(100), 191.0859(6), 219.0806(23) | neolitsine isomer |
| 67 | 18.18 | 324.1230 | 324.1234 | 1.16 | $[C_{19}H_{17}NO_4+H]^+$ | $MS^2$[324]: 264.1018(100), 58.0660(53), 233.0827(17), 263.0926(17), 265.1074(16) | N-acetylanonaine |
| 68 | 18.53 | 292.0968 | 292.0972 | 1.12 | $[C_{18}H_{13}NO_3 +H]^+$ | $MS^2$[292]:277.1039(100), 248.0712(8), 264.1024(13) | neolitsine isomer |
| 69 | 18.81 | 292.0968 | 292.0971 | 0.89 | $[C_{18}H_{13}NO_3+H]^+$ | $MS^2$[292]:264.1018(100), 248.0702(8), 277.0734(48) | lysicamine isomers |
| 70 | 19.08 | 338.1386 | 338.1387 | 0.13 | $[C_{20}H_{19}NO_4+H]^+$ | $MS^2$[338]: 307.1201(100), 279.1258(4), 308.1265(10), 323.1153(16) | lysicamine isomers |
| | | | | | | | sinomendine |

* identified by comparison with standards.
